# Evaluation of [^68^Ga]Ga-NODAGA-RGD for PET Imaging of Rat Autoimmune Myocarditis

**DOI:** 10.3389/fmed.2021.783596

**Published:** 2021-12-15

**Authors:** Arghavan Jahandideh, Mia Ståhle, Jenni Virta, Xiang-Guo Li, Heidi Liljenbäck, Olli Moisio, Juhani Knuuti, Anne Roivainen, Antti Saraste

**Affiliations:** ^1^Turku PET Centre, University of Turku, Turku, Finland; ^2^Turku Center for Disease Modeling, University of Turku, Turku, Finland; ^3^Turku PET Centre, Turku University Hospital, Turku, Finland; ^4^Heart Center, Turku University Hospital and University of Turku, Turku, Finland

**Keywords:** angiogenesis, α_v_β_3_ integrin, inflammation, positron emission tomography, myocarditis

## Abstract

The ^68^Gallium-labeled 1,4,7-triazacyclononane-1-glutaric acid-4,7-diacetic acid conjugated radiolabelled arginine-glycine-aspartic acid peptide ([^68^Ga]Ga-NODAGA-RGD) is a positron emission tomography (PET) tracer binding to cell surface receptor α_v_β_3_ integrin that is upregulated during angiogenesis and inflammation. We studied whether α_v_β_3_ targeting PET imaging can detect myocardial inflammation in a rat model of autoimmune myocarditis. To induce myocarditis, rats (*n* = 8) were immunized with porcine cardiac myosin in complete Freund's adjuvant on days 0 and 7. Control rats (*n* = 8) received Freund's adjuvant alone. On day 21, *in vivo* PET/CT imaging with [^68^Ga]Ga-NODAGA-RGD followed by *ex vivo* autoradiography and immunohistochemistry were carried out. Inflammatory lesions were detected histologically in the myocardium of 7 out of 8 immunized rats. *In vivo* PET images showed higher [^68^Ga]Ga-NODAGA-RGD accumulation in the myocardium of rats with inflammation than the non-inflamed myocardium of control rats (SUV_mean_ 0.4 ± 0.1 vs. 0.1 ± 0.02; *P* = 0.00006). *Ex vivo* autoradiography and histology confirmed that [^68^Ga]Ga-NODAGA-RGD uptake co-localized with inflammatory lesions containing α_v_β_3_ integrin-positive capillary-like structures. A non-specific [^68^Ga]Ga-DOTA-(RGE)_2_ tracer showed 76% lower uptake than [^68^Ga]Ga-NODAGA-RGD in the inflamed myocardium. Our results indicate that α_v_β_3_ integrin-targeting [^68^Ga]Ga-NODAGA-RGD is a potential PET tracer for the specific detection of active inflammatory lesions in autoimmune myocarditis.

## Introduction

Myocarditis is defined as myocardial inflammation characterized by immune cell infiltration and myocyte necrosis, resulting from infectious or non-infectious initiators (1). Clinical sequelae of myocarditis include conduction disturbances, ventricular arrhythmias, and inflammatory cardiomyopathy defined as myocarditis associated with cardiac dysfunction (1).

Positron emission tomography (PET)/CT with 2-deoxy-2-[^18^F]fluoro-*D*-glucose ([^18^F]FDG) is a sensitive non-invasive imaging technique for the detection of myocardial inflammation. While cardiac [^18^F]FDG PET has shown good accuracy in the detection of cardiac inflammation, the non-specific physiological uptake of the [^18^F]FDG in the myocardium may limit its specificity and usefulness in monitoring active disease (2, 3). Recent studies have provided evidence that more specific identification of myocardial inflammatory lesions could be possible with alternative tracers (4–10).

Angiogenesis, which is known as the formation of new capillaries from pre-existing vessels, plays a role in the pathogenesis of various human diseases. Inflammatory cytokines are one of the key stimulants of angiogenesis (11). Studies have shown increased angiogenesis in cancer (12), ischaemic heart disease (13) as well as many inflammatory diseases, such as rheumatoid arthritis (14, 15), diabetic retinopathy (16), psoriasis (17), pulmonary sarcoidosis or fibrosis (18, 19), and atherosclerosis (20, 21).

The α_v_β_3_ integrin is a cell membrane glycoprotein receptor that is highly expressed on angiogenic endothelial cells, mediates their adhesion to the extracellular matrix, and functions as a regulator of angiogenesis (11, 22, 23). Radiolabelled arginine-glycine-aspartic acid (RGD) derivatives targeting α_v_β_3_ integrin have been investigated as tracers to image angiogenesis in cancer, inflammation, and cardiovascular diseases (24). We have previously demonstrated that cardiac PET with radiolabelled RGD peptides, such as ^68^Gallium-labeled 1,4,7-triazacyclononane-1-glutaric acid-4,7-diacetic acid conjugated RGD peptide ([^68^Ga]Ga-NODAGA-RGD), can be used to detect α_v_β_3_ integrin expression after ischemic myocardial injury (25–27). However, α_v_β_3_ integrin targeted imaging has not been investigated in myocarditis. We hypothesized that myocarditis-associated cardiac inflammation is associated with enhanced expression of α_v_β_3_ integrin in angiogenic endothelial cells and activated macrophages providing a target for imaging.

Here, we studied the expression of α_v_β_3_ integrin in myocardial inflammatory lesions and evaluated the feasibility of detecting myocardial inflammation in a rat model of autoimmune myocarditis using α_v_β_3_ targeted [^68^Ga]Ga-NODAGA-RGD PET.

## Materials and Methods

### Animal Model and the Study Protocol

All animal experiments were approved by the National Animal Experiment Board in Finland and the Regional State Administrative Agency for Southern Finland. Autoimmune myocarditis was induced in rats as previously described (4). Briefly, eight male Lewis rats aged 6 to 8 weeks (weight in g, 287 ± 17) were immunized with subcutaneous injections of 5 mg/ml pig cardiac myosin (M0531; Sigma Aldrich) in an equal volume of complete Freund's adjuvant supplemented with 1 mg/ml heat-killed and dried *mycobacterium tuberculosis* (F5881; Sigma Aldrich) into the hock of the left foot twice 7 days apart (days 0 and 7). To enhance the immunization effect, intraperitoneal injection of 250 ng/ml pertussis toxin (P2980; Sigma Aldrich) was also made on day 0. Eight male control Lewis rats (weight in g, 293.2 ± 23) received the adjuvant alone into the hock of the left foot. All procedures were carried out under isoflurane anesthesia (1.5–2.5%) and buprenorphine (0.03 mg/kg) was being administered two times a day for 2 days after immunization for analgesia.

Positron emission tomography (PET) imaging with [^68^Ga]Ga-NODAGA-RGD was performed 3 weeks after the first immunization (day 21). To visualize the myocardium, contrast-enhanced CT or [^18^F]FDG PET imaging was carried out. At 100 min after imaging with [^68^Ga]Ga-NODAGA-RGD, rats were euthanized and the heart and other organs were excised, weighed, and analyzed for the biodistribution of [^68^Ga]Ga-NODAGA-RGD utilizing a gamma counter (Triathler 3″; Hidex, Turku, Finland) (4). The heart was frozen and sliced into serial transverse 20 and 8 μm cryosections at 1 mm intervals from base to apex for autoradiography, histology, and immunostainings.

### Radiotracer

^68^Gallium-labeled 1,4,7-triazacyclononane-1-glutaric acid-4,7-diacetic acid conjugated RGD peptide ([^68^Ga]Ga-NODAGA-RGD) was synthesized according to previously described procedures (27). The radiochemical purity was ≥ 99% and the molar activity was 18 ± 7.3 GBq/μmol (*n* = 9). A non-targeted peptide [^68^Ga]Ga-DOTA-E[c(RGEfK)]_2_, where E[c(RGEfk)]_2_ = glutamic acid-[cyclo(arginyl-glycyl-glutamic acid-*D*-phenylalanine-lysine)], was used as a negative control as previously described (28).

### *In vivo* PET/CT Imaging

The rats were imaged using a small animal PET/CT device (Inveon Multimodality; Siemens Medical Solutions) after [^68^Ga]Ga-NODAGA-RGD injection under isoflurane anesthesia as previously described (4). The rats were injected with 50.8 ± 2.8 MBq [^68^Ga]Ga-NODAGA-RGD intravenously and either a 10 min static PET acquisition starting at 60 min post-injection (*n* = 4 immunized rats and *n* = 8 controls) or a 90 min dynamic PET acquisition starting at the time of injection (6 × 10 s, 4 × 60 s, 5 × 300 s, and 6 × 600 s frames, *n* = 4 immunized rats) was performed to study the kinetics of the tracer.

In order to localize the myocardium, a high-resolution CT was acquired in all animals immediately after the [^68^Ga]Ga-NODAGA-RGD PET injection 300 μL intravascular iodinated contrast agent (eXia™ 160XL; Binitio Biomedical Inc.) as described earlier (4). In addition to CT, a 40 min static [^18^F]FDG (38.5 ± 2.9 MBq) PET acquisition starting at 20 min post-injection was carried out in 11 rats (*n* = 6 immunized rats and *n* = 5 controls) on day 20 to facilitate localization of the myocardium.

[^68^Ga]Ga-NODAGA-RGD PET images were co-registered with CT or [^18^F]FDG images using Carimas 2.9 software (Turku PET Center, Turku, Finland), and regions of interest (ROI) with uniform size were defined in the left ventricle (LV) myocardium in [^68^Ga]Ga-NODAGA-RGD images. In immunized rats, ROIs were drawn in myocardial areas roughly corresponding to inflammatory lesions in histology. In controls rats, ROIs were drawn in similar areas as in immunized rats. The uptake is expressed as mean standardized uptake value (SUV_mean_) and was compared between inflammatory lesions of immunized rats and the non-inflamed myocardium of control rats. Blood pool activity was measured as the average of ROIs drawn in the LV cavity and inferior vena cava. Additional ROIs were drawn in the lung, liver, kidney, and foreleg muscle. Decay-corrected time-activity curves (TACs) were extracted from dynamic image data.

### *Ex vivo* Autoradiography, Histology, and Immunostainings

For histology, 20 μm cryosections were stained with H&E. For immunohistochemistry, adjacent serial 8 μm cryosections were stained with a monoclonal mouse anti-rat CD68 (1:1000, MCA341GA, Bio-Rad, Hercules, CA, USA) to detect macrophages, a monoclonal mouse anti-rat CD61 antibody (dilution 1:500, MCA1773, Bio-Rad, Hercules, CA, USA) to detect integrin β3 chain, a polyclonal rabbit anti-rat CD31 antibody (1:200, NB100-2284, Novus Biologicals, Centennial, CO, USA) to detect endothelial cells, and monoclonal mouse anti- α-smooth muscle actin (α-SMA) antibody (1:12,000, A5228-200, Sigma-Aldrich, St. Louis, MO, USA) to detect myofibroblasts.

Uptake of [^68^Ga]Ga-NODAGA-RGD in the inflamed and non-inflamed myocardium was analyzed by *ex vivo* digital autoradiography of 20 μm tissue sections as previously described (4). Multiple ROIs were drawn in the inflamed and non-inflamed myocardium in co-registered autoradiographs and images of H&E stainings of the same sections using TINA™ 2.10f software (Raytest Isotopenmessgeräte GmbH., Straubenhardt, Germany). Inflamed myocardium was defined as the presence of inflammatory cell infiltrate and signs of myocyte necrosis in the H&E-stained section. Non-inflamed myocardium was defined as the absence of both inflammations in the H&E-stained section and CD68 positive macrophages in a serial tissue section. The whole area of histologically inflamed myocardium was sampled, whereas the non-inflamed myocardium was sampled using multiple, uniformly sized ROIs. The results are as average photo-stimulated luminescence per square millimeter (PSL/mm^2^).

### Studies With Non-specific [^68^Ga]Ga-DOTA-E[c(RGEfK)]_2_ Peptide

To study the specificity of [^68^Ga]Ga-NODAGA-RGD uptake in cardiac inflammatory lesions, uptake of a non-specific control peptide [^68^Ga]Ga-DOTA-E[c(RGEfK)]_2_ was analyzed in three immunized rats (weight in g, 310.3 ± 6.6). The rats were injected with 52.4 ± 0.8 MBq [^68^Ga]Ga-DOTA-E[c(RGEfK)]_2_ via the tail vein and euthanized at 90 min post-injection. Then, tracer biodistribution was analyzed and *ex vivo* autoradiography of 20 μm cryosections of the myocardium was performed as described above.

### Statistical Analysis

Formal power calculation was not performed for this exploratory study. However, uptake of [^68^Ga]DOTA-RGD in healthy myocardium (SUV 0.19 ± 0.02) in a previous study (26) indicated that it was possible to detect at least 20% difference in tracer uptake with a sample size of 6 rats at 90% probability and the alpha value of 0.05. All data are expressed as mean ± *SD*. Statistical analysis was performed with GraphPad Prism 6 Software. Unpaired or paired Student's *t*-test was used for single comparisons between two groups with normally distributed data. The statistical significance threshold was *P* < 0.05.

## Results

### Histology

**Figure 1:**
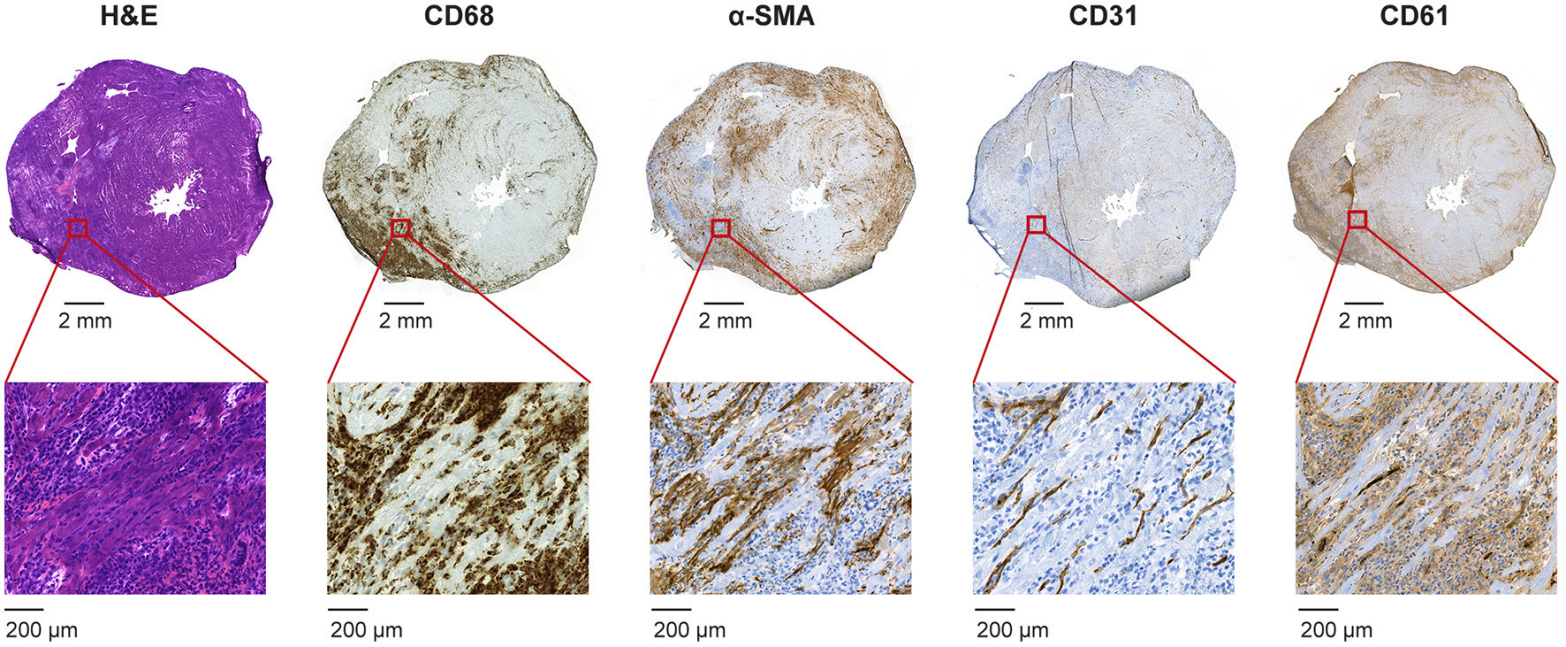
Histology of myocardial inflammatory lesions in a cross-section of the heart in an immunized rat. Hematoxylin and eosin (H&E) staining shows some diffuse inflammation and a large inflammatory lesion in the inferior wall of the left ventricle extending to the right ventricular wall characterized by infiltrated inflammatory cells and myocyte necrosis. Immunostaining (brown color) shows dense infiltrates of CD68-positive macrophages, some α-smooth muscle actin (α-SMA)-positive myofibroblasts as well as a dense capillary network of CD31-positive endothelial cells. Staining with CD61 antibody indicates that some of the capillaries express integrin β_3_.

Histological analysis showed that of the eight immunized rats, seven (78%) developed wide-spread cardiac inflammatory lesions, whereas none of the eight control rats developed any inflammatory lesions (Figure 1).

The inflammatory lesions were characterized by intense inflammatory cell infiltration and myocyte necrosis. Consistent with our previous findings in this model (4), the immunohistochemical staining revealed that CD68-positive macrophages are the most abundant inflammatory cells in the lesions. Positive α-SMA staining indicates the presence of myofibroblasts and CD31 positive staining shows the presence of capillary network within the inflammatory lesions (Figure 1). CD61 staining co-localized in the same areas with CD31-positive staining and there were capillary-like structures showing positive staining (Figure 1).

### *In vivo* PET/CT Imaging

**Figure 2:**
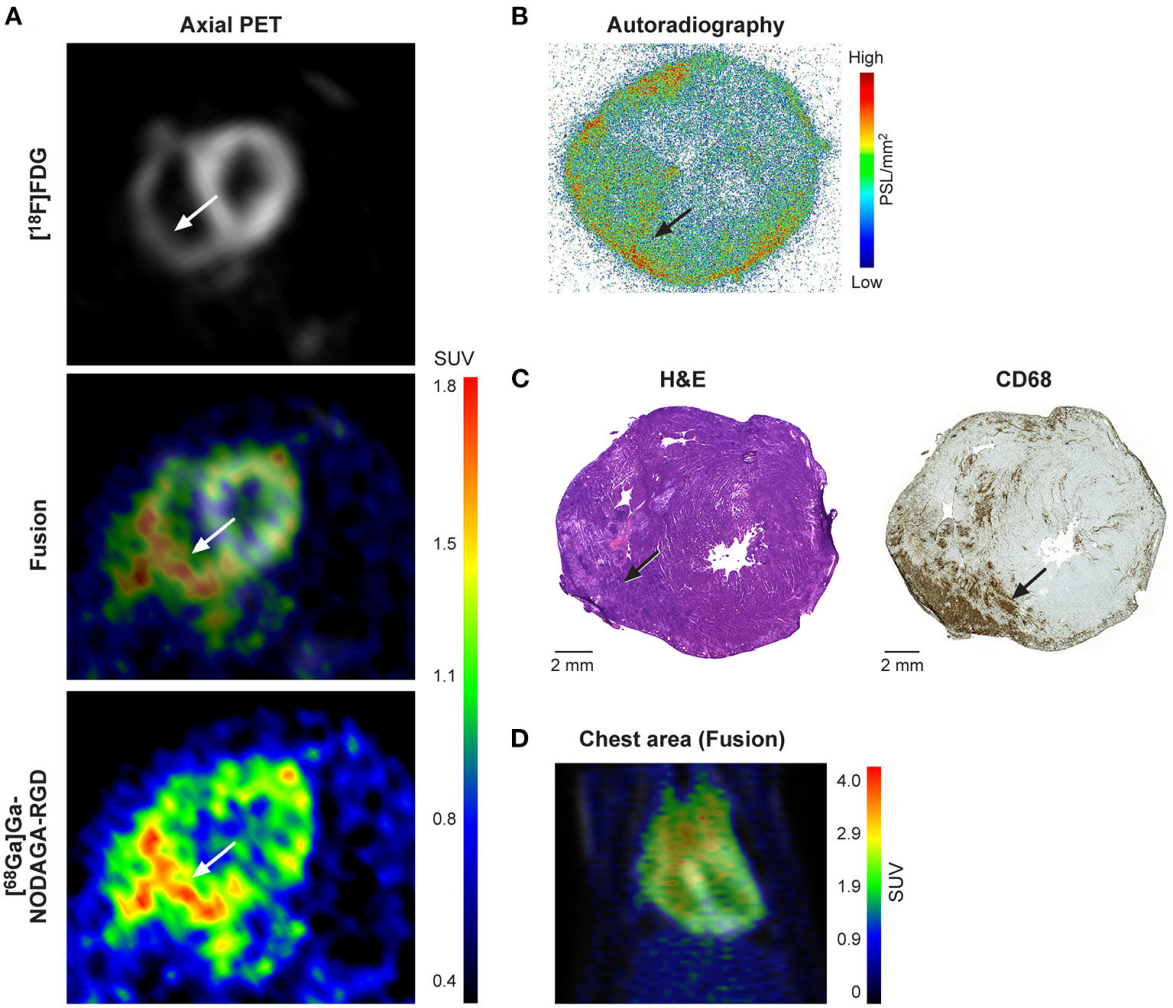
**(A)** *In vivo* PET images with [^18^F]FDG and [^68^Ga]Ga-NODAGA-RGD tracers in an immunized rat. PET images at 60-80 min show [^68^Ga]Ga-NODAGA-RGD uptake (white arrows) predominantly in the inferior wall of the left ventricle extending to the right ventricular wall. **(B)** *Ex vivo* autoradiography shows myocardial [^68^Ga]Ga-NODAGA-RGD uptake mainly in the inferior wall of the left ventricle extending to the right ventricular wall that co-localizes with an inflammatory myocardial lesion seen on the same and adjacent histological sections stained with hematoxylin and eosin (H&E) or antibodies against CD68 (macrophages) **(C)**. Note some tracer uptake and inflammation also in the epicardial myocardium close to the pericardium. Outside inflammatory lesions, the myocardium shows very low uptake. **(D)** *In vivo* PET image with [^18^F]FDG (gray) and [^68^Ga]Ga-NODAGA-RGD (rainbow) tracers showing the chest area in an immunized rat.

*In vivo* [^68^Ga]Ga-NODAGA-RGD PET/CT images showed visually increased tracer uptake in the myocardium of all immunized rats with histological inflammation (*n* = 7), while there was no visible tracer uptake in the myocardium of the immunized rat without inflammation (*n* = 1) or control rats (*n* = 8) (Figure 2).

**Figure 3:**
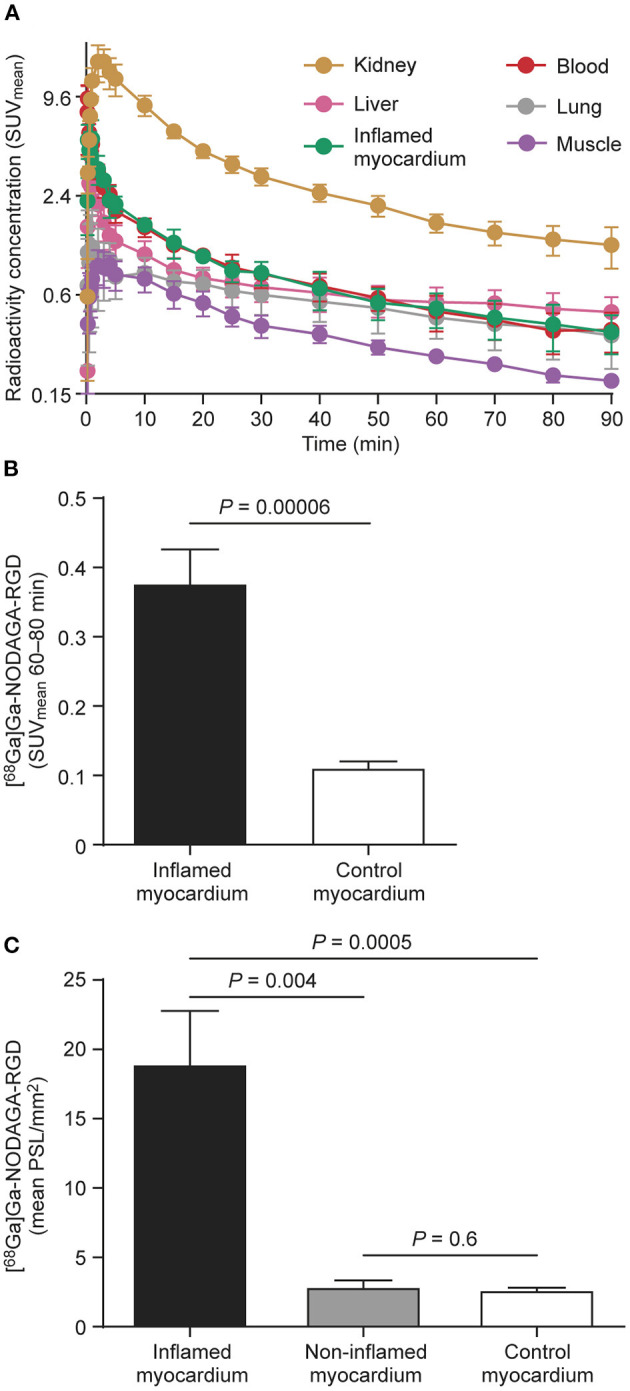
**(A)** Time-activity curves of [^68^Ga]Ga-NODAGA-RGD from rats with autoimmune myocarditis (*n* = 4) shows that tracer uptake (mean standardized uptake value, SUV_mean_) remains numerically slightly higher in inflamed myocardium than in blood. Bars indicate standard deviation. Note that the y-axis is on a logarithmic scale. **(B)** The average myocardial [^68^Ga]Ga-NODAGA-RGD uptake (SUV_mean_) 60–80 min after injection significantly increased in the inflamed myocardium (*n* = 7) as compared to the myocardium of control rats (*n* = 8). **(C)** Bars demonstrate [^68^Ga]Ga-NODAGA-RGD uptake by *ex vivo* autoradiography as mean photo-stimulated luminescence per square millimeter (PSL/mm^2^) in inflamed myocardium (*n* = 7), non-inflamed myocardium of immunized rats (*n* = 7), and myocardium of control rats (*n* = 8). Values are mean ± *SD*; unpaired *t*-tests for comparisons of inflamed and control myocardium, and paired *t*-tests for comparisons of inflamed and non-inflamed myocardium.

Analysis of the kinetics of [^68^Ga]Ga-NODAGA-RGD uptake based on TACs demonstrated that tracer uptake in the inflamed myocardium was most stable after 30 min post-injection (Figure 3A).

The uptake of the [^68^Ga]Ga-NODAGA-RGD at 60–80 min post-injection was significantly higher in the inflamed myocardium compared to the non-inflamed myocardium of control rats (SUV_mean_, 0.4 ± 0.1 vs. 0.1 ± 0.02; *P* = 0.00006, Figure 3B). The blood SUV_mean_, was 0.3 ± 0.1 (*p* = 0.09 vs. inflamed myocardium) and the uptake ratio between inflamed myocardium and blood was 1.4 ± 0.5.

### *Ex vivo* Autoradiography and Biodistribution

The results of [^68^Ga]Ga-NODAGA-RGD biodistribution at 100 min are shown in Table 1. Tracer uptake was higher in the myocardium than in blood in both immunized and control rats (*P* < 0.01). Compared with control rats, tracer accumulation was higher in the hearts and lymph nodes (mediastinal and axillary) in the immunized rats. Notably, in addition to the heart, inflammation was evident in the lymph nodes of immunized rats by histology (Supplementary Figure 1).

**Table 1:** *Ex vivo* biodistribution of [^68^Ga]Ga-NODAGA-RGD in rats at 100 min after intravenous injection.

	**Immunized rats**	**Control rats**	***P*-value**
	**(*n* = 7)**	**(*n* = 8)**	
Blood	0.16 ± 0.10	0.14 ± 0.05	0.80
Heart	0.34 ± 0.10	0.21 ± 0.08	0.029^*^
Intestine without content	1.44 ± 0.32	2.61 ± 1.37	0.07
Kidney	2.24 ± 0.87	3.19 ± 1.59	0.23
Liver	0.62 ± 0.17	1.12 ± 0.69	0.12
Lung	0.64 ± 0.21	0.89 ± 0.47	0.26
Lymph node	0.72 ± 0.19	0.39 ± 0.72	0.05^*^
Muscle	0.11 ± 0.03	0.15 ± 0.07	0.29
Plasma	0.32 ± 0.16	0.25 ± 0.10	0.41
Pancreas	0.30 ± 0.08	0.58 ± 0.45	0.18
Spleen	0.80 ± 0.30	1.79 ± 1.42	0.14
Thymus	0.43 ± 0.15	0.40 ± 0.20	0.80
Urine	32.98 ± 6.30	34.42 ± 5.73	0.70
White adipose tissue	0.10 ± 0.03	0.09 ± 0.05	0.84

*The results are expressed as SUVs (mean ± SD)*.

* *A statistically significant difference based on Student's t-test*.

Autoradiography showed focally increased [^68^Ga]Ga-NODAGA-RGD uptake in the LV myocardium of immunized rats colocalizing with histological inflammatory lesions. Tracer uptake was low in the non-inflamed myocardium of immunized rats or myocardium of controls. Quantitatively, the average [^68^Ga]Ga-NODAGA-RGD uptake in the inflamed area was 6.7 ± 3.0-fold higher than the uptake in the non-inflamed area of immunized rats (19 ± 9.3 vs. 2.9 ± 1.1 PSL/mm^2^; *P* = 0.004) and 7.1 ± 3.5-fold higher than controls (2.7 ± 0.4 PSL/mm^2^ *n* = 8; *P* = 0.0005; Figure 3C).

### Studies With Non-specific [^68^Ga]Ga-DOTA-E[c(RGEfK)]_2_

*Ex vivo* autoradiography showed that uptake of non-specific [^68^Ga]Ga-DOTA-E[c(RGEfK)]_2_ in histologically confirmed inflammatory lesions of three immunized rats was 76% lower as compared to [^68^Ga]Ga-NODAGA-RGD in inflammatory lesions of seven immunized rats (4.5 ± 0.6 vs. 19 ± 9.3 PSL/mm^2^; *P* = 0.04). The average [^68^Ga]Ga-DOTA-E[c(RGEfK)]_2_ uptakes in the inflamed area was higher than the uptake in the non-inflamed area of those three immunized rats (4.5 ± 0.6 vs. 0.9 ± 0.3 PSL/mm^2^; *P* = 0.004) but lower than the uptake of [^68^Ga]Ga-NODAGA-RGD in the inflamed area.

## Discussion

We demonstrated that [^68^Ga]Ga-NODAGA-RGD shows specific uptake in inflammatory myocardial lesions in a rat model of autoimmune myocarditis. *Ex vivo* autoradiography showed more than six times higher [^68^Ga]Ga-NODAGA-RGD uptake in inflammatory lesions of immunized rats as compared with the non-inflamed myocardium. The uptake was significantly higher than that of a non-specific tracer indicating a high degree of specific binding. The [^68^Ga]Ga-NODAGA-RGD signal was modestly, but significantly higher in the inflamed than non-inflamed myocardium also in *in vivo* PET images.

Rat model of autoimmune myocarditis is a well-established model of myocarditis, which has been shown to represent features of human giant cell myocarditis eventually progressing to dilated cardiomyopathy (29–31). The model is characterized by the high prevalence of macrophages in the inflammatory lesions (4). We provided evidence that α_v_β_3_ integrin is expressed in this model at 21 d after first immunization when the peak in acute inflammation in this model occurs (29, 30). α_v_β_3_ integrin mediates angiogenesis and plays a role in the regulation of macrophage responses during inflammation (21, 22, 32, 33). We found a capillary network within the inflammatory lesions and capillary-like structures that were stained with integrin β_3_ antibodies. Thus, we suggest that [^68^Ga]Ga-NODAGA-RGD uptake is associated with the presence of angiogenesis in the inflammatory lesions similar to that observed after an ischemic myocardial injury (24–26). However, it has been demonstrated that α_v_β_3_ integrin can also be expressed by macrophages that were also present in inflammatory lesions (21, 32). Consistent with the interplay between angiogenesis and inflammation, our results indicate that imaging of α_v_β_3_ integrin expression is a potential target for imaging myocardial inflammatory activity in myocarditis.

Imaging of α_v_β_3_ expression in inflamed myocardium by [^68^Ga]Ga-NODAGA-RGD offers a potential approach for the non-invasive assessment of myocarditis. Our study revealed that [^68^Ga]Ga-NODAGA-RGD shows specific uptake in the inflamed myocardium with increased α_v_β_3_ integrin expression detectable by *in vivo* PET imaging. This was confirmed by injection of a non-specific tracer [^68^Ga]Ga-DOTA-E[c(RGEfK)]_2_, which showed 76% lower uptake. Our results also suggest that [^68^Ga]Ga-NODAGA-RGD PET provides a relatively high contrast between inflamed and non-inflamed myocardium (6.7 ± 3.0) that is similar to or even higher than with other tracers evaluated in the same animal model, such as [^18^F]-FOL (7.8 ± 1.4) (4), [^18^F]FDG (3.4 ± 0.7) (5), or [^11^C]methionine (2.1 ± 0.2) (5). We have previously shown in a rat model of myocardial infarction that measurement of SUV in static images showed comparable results to kinetic modeling of distribution volume of [^68^Ga]DOTA-RGD uptake simplifying *in vivo* analysis (26). The fact that [^68^Ga]Ga-NODAGA-RGD, like other α_v_β_3_ targeted tracers, have been previously used in humans, may facilitate clinical studies on this approach also in the setting of myocarditis (24, 34).

The biodistribution result showed increased accumulation of [^68^Ga]Ga-NODAGA-RGD in the lymph nodes of immunized rats that is most likely attributed to inflammation in these tissues (Supplementary Figure 1). This is consistent with the common involvement of mediastinal lymph nodes in inflammatory cardiac disease (1).

There are some limitations in our experimental study. One is that we were not able to directly compare uptake of [^68^Ga]Ga-NODAGA-RGD in inflamed myocardium with that of [^18^F]FDG, because of suppression of physiological [^18^F]FDG uptake in the myocardium was not successful in our model. Another limitation of our study is a relatively weak signal of the inflamed myocardium compared to the adjacent liver in *in vivo* PET images with [^68^Ga]Ga-NODAGA-RGD. Due to weak signal, the low resolution of PET images, and diffuse inflammatory lesions, comparison of [^68^Ga]Ga-NODAGA-RGD uptake between inflamed and adjacent non-inflamed myocardium of immunized rats was not feasible in *in vivo* images, but was evident by *ex vivo* autoradiography. The exact role of α_v_β_3_ integrin and angiogenesis in myocarditis is currently uncertain and therefore, our results should be considered only as an initial proof that [^68^Ga]Ga-NODAGA-RGD imaging can detect myocardial inflammatory lesions.

In conclusion, [^68^Ga]Ga-NODAGA-RGD is specifically taken up in myocardial inflammatory lesions indicating increased α_v_β_3_ integrin expression in a rat model of autoimmune myocarditis. Targeted PET imaging of α_v_β_3_ integrin is a potential approach for the detection of active myocardial inflammation.

## Data Availability Statement

The original contributions presented in the study are included in the article/Supplementary Material, further inquiries can be directed to the corresponding author/s.

## Ethics Statement

The animal study was reviewed and approved by the National Animal Experiment Board in Finland and the Regional State Administrative Agency for Southern Finland.

## Author Contributions

AS, AR, and JK contributed to study design, supervision, data analysis, and funding acquisition. AJ contributed to data collection, data analysis, and wrote the first draft of the manuscript. X-GL, MS, JV, HL, and OM contributed to data collection. All authors contributed to the writing of the manuscript and approved the submitted version of the manuscript.

## Funding

This study was financially supported by grants from the Academy of Finland (310136 and 343152), Jane and Aatos Erkko Foundation, Finnish Foundation for Cardiovascular Research, the Finnish Medical Foundation, Sigrid Jusélius Foundation, and State Research Funding from the Hospital District of Southwest Finland.

## Conflict of Interest

AS received consultancy or speaker fees from Amgen, Astra Zeneca, Boehringer Ingelheim, Abbott, and Bayer not related to the present study. JK received consultancy fees from GE Healthcare and AstraZeneca and speaker fees from GE Healthcare, Bayer, and Lundbeck. Boehringer-Ingelheim and Merck, outside of the submitted work. The remaining authors declare that the research was conducted in the absence of any commercial or financial relationships that could be construed as a potential conflict of interest.

## Publisher's Note

All claims expressed in this article are solely those of the authors and do not necessarily represent those of their affiliated organizations, or those of the publisher, the editors and the reviewers. Any product that may be evaluated in this article, or claim that may be made by its manufacturer, is not guaranteed or endorsed by the publisher.

## Supplementary Material

Supplementary Figure 1 Histology of the left axillary lymph node in an immunized rat stained with hematoxylin and eosin (H&E) or antibodies against CD68 (macrophages).
