# Supplementary figures and images for: Evaluation of [68Ga]Ga-NODAGA-RGD for PET Imaging of Rat Autoimmune Myocarditis
Source: Front Med (Lausanne). 2021 Dec 15;8:783596. doi: 10.3389/fmed.2021.783596 (PMC8714834; doi:10.3389/fmed.2021.783596)

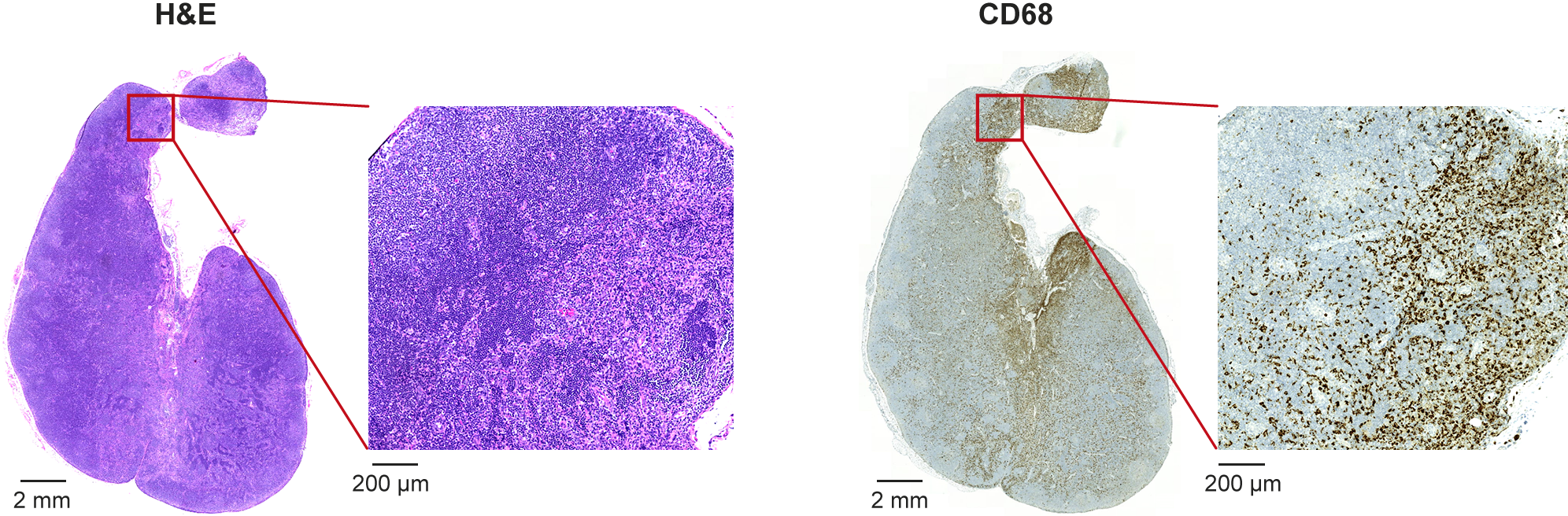

Supplement: Supplementary Figure 1 — Histology of the left axillary lymph node in an immunized rat stained with hematoxylin and eosin (H&E) or antibodies against CD68 (macrophages). [file Image_1.TIF]
